# *Talaromyces variabilis* interferes with *Pythium aphanidermatum* growth and suppresses *Pythium*-induced damping-off of cucumbers and tomatoes

**DOI:** 10.1038/s41598-019-47736-x

**Published:** 2019-08-02

**Authors:** Boshra A. Halo, Rashid A. Al-Yahyai, Sajeewa S. N. Maharachchikumbura, Abdullah M. Al-Sadi

**Affiliations:** Department of Crop Sciences, College of Agricultural and Marine Sciences, Sultan Qaboos University, PO Box 34, Al-Khoud, 123 Oman

**Keywords:** Fungi, Plant sciences

## Abstract

Pythium-induced damping-off disease is a major disease limiting cucumber and tomato production in different parts of the world. The current study investigated the efficiency of *Talaromyces variabilis* and its bioactive metabolites in suppressing *Pythium*-induced damping-off of cucumbers and tomatoes. *T*. *variabilis* inhibited the *in vitro* growth of *P*. *aphanidermatum* in solid and liquid media. In addition, abnormalities in *P*. *aphanidermatum* hyphae were observed as a result of *T*. *variabilis*. Extracts from *T*. *variabilis* induced cellular leakage and suppressed oospore production of *P*. *aphanidermatum*. Biochemical analyses of *T*. *variabilis* metabolites showed that *T*. *variabilis* produces glucanase, cellulase and siderophores, suggesting the contribution of these metabolites in the inhibition of *P*. *aphandermatum* growth and in hyphal abnormalities. Treating cucumber seeds with spore and mycelial suspension of *T*. *variabilis* isolates led to a significant improvement in the seedling survival of *P*. *aphanidermatum*-inoculated seedlings from 18 to 52% (improvement by 34%) for isolate 48 P and from 30–66% (improvement by 36%) for isolate 28 R. Similarly, treating tomato seeds with spore and mycelial suspension of *T*. *variabilis* isolates led to a significant improvement in the seedling survival of *P*. *aphanidermatum*-inoculated seedlings from 7 to 36% (improvement by 29%) for isolate 28 R and from 20 to 64% (improvement by 44%) for isolate 48 P. Differences in the percent improvement in seedling survival between experiments may be related to difference in the efficacy of the two different isolates or their interaction with the hosts and pathogen. The use of *T*. *variabilis* in the biocontrol of *Pythium*-induced diseases may offer alternatives to the currently used chemical control.

## Introduction

Soil-borne pathogens represent a major challenge to crops worldwide. Diseases and loses caused by soil-borne pathogens vary from one place and crop to another depending on the pathogen, environmental conditions and management strategies. *Pythium* species are a major problem worldwide especially in vegetable crops. *Pythium*-induced damping-off and root diseases of cucurbits and tomatoes can result in losses of up to 100%^1,2^. Diseases in these crops are caused by various *Pythium* species, the most common of which is *P*. *aphanidermatum*^3,4^.

Damping-off and root diseases of vegetable crops can be managed using chemical treatments (e.g. Mefenoxam, Hymexazol and Captan)^5,6^. Since chemical control has several negative effects on the environment and humans, other environmentally safe methods have been used or developed, including the use of solarization^7^. Biological control, which depends on microorganisms, including endophytes, is better than using synthetic chemical fungicides, because of their hazards to the environment as well as the potential development of resistance to fungicides^8^.

Endophytic microorganisms reside inside plant tissues and have multiple benefits to their hosts and environments including mineral solubilization^9,10^, phytohormones production^11^, phytoremediation of heavy metals^12,13^ and disease suppression^14,15^.

Several endophytic fungi are used as biocontrol agents against plant disease such as *Botryosphaeria ribis*, *Trichoderma* sp. and *Aspergillus terreus*^14,16^. *Talaromyces* species have multiple benefits for plants; they have been used as biocontrol agents against several plant pathogen^17,18^. In Oman, little attention has been given to finding biocontrol agents against soil borne diseases.

There are several mechanisms that microbes use to promote disease stress tolerance in plants including hydrolytic enzymes production^19^, siderophores production^20^ and hydrogen cyanide synthesis^21^. Generally, endophytes reduce pathogen effects in plants immediately after infection by promoting plant stress response systems^22,23^. Elucidating the ways by which biocontrol agents affect other pathogens is helpful in coming up with effective management strategies for pathogens.

During a recent study in Oman, two *Talaromyces* isolates with potential biocontrol activities were isolated. This study aimed at investigating the suppressive effects of these isolates against growth, and spore production by *Pythium aphanidermatum*, the potential metabolites involved in the inhibition, and the potential biocontrol activities of the isolates against Pythium damping-off of cucumbers and tomatoes.

Knowledge in these areas could help come up with effective biocontrol agents against soil borne disease affecting crops in Oman.

## Results

### Identification of *Talaromyces* isolates

**Figure 1:**
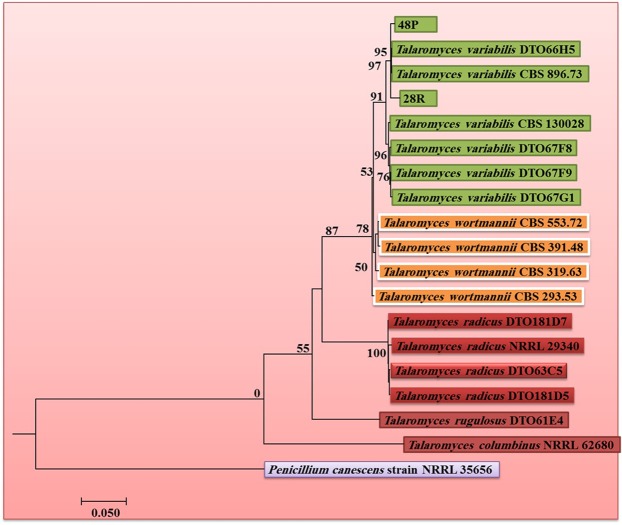
Maximum likelihood tree for the analysis *Talaromyces* spp. based on the combined ITS, TUB, and CMD gene regions. RAxML bootstrap support values above 50% are given at the nodes. The tree is rooted with *Penicillium canescens* (NRRL 62680). 48 P and 28 R are the isolates used in this study.

The combined ITS, TUB and CMD dataset comprises 18 isolates of *Talaromyces*. Phylogenetic analysis showed that isolates 48 P and 28 R belong to *T*. *variabilis* (Fig. 1).

### Antagosnistic effect of *T*. *variabilis* isolates against *P*. *aphanidermatum*

Both *T*. *variabilis* isolates showed antagonistic activity against *P*. *aphanidermatum* in PDA medium (Table 1). *T*. *variabilis* isolates 48 P and 28 R produced inhibition zones of 8.5 mm and 6.25 mm, respectively.

**Table 1:** Antagosnistic effects of *T*. *variabilis* isolates against *P*. *aphanidermatum*.

Treatments	Inhibition zone (mm)	Time taken to fill plate (days)	Number of produced spores	Extracellular conductivity: 24 h- 0 min (mV)	Glucanase activity (nmoles hr^−1^ ml^−1^)	Cellulase activity: μmol/min/ml
Control	0 ± 0c	NA	56.33 ± 7.5a	0.86 ± 0.95b	NA	NA
48 P	8.5 ± 1.29a	13.5 ± 0.58a	8 ± 1.73b	21.56 ± 1.67a	8.09 ± 0.15a	2.12 ± 1.09a
28 R	6.25 ± 0.96b	15.5 ± 2.08a	8.66 ± 3.78b	13.66 ± 5.16a	8.21 ± 0.04a	0.43 ± 0.09b

Values with the same letters in the same column are not significantly different from each other (Duncan test, P > 0.05 for 3 treatments, ANOVA Test, P > 0.05 for 2 treatments), the values represent the mean ± SD. NA (not applicable).

The second experiment was conducted in PDA plates to observe the antagonistic effect of *T*. *variabilis* isolates over time. *P*. *aphanidermatum* ceased its growth. However, *T*. *variabilis* isolates 48 P and 28 R continued to grow towards *P*. *aphanidermatum* and filled the plate after 13.5 days and 15.5 days, respectively (Table 1).

### Effect of culture filtrates of *T*. *variabilis* on *P*. *aphanidermatum* growth and oospore production

**Figure 2:**
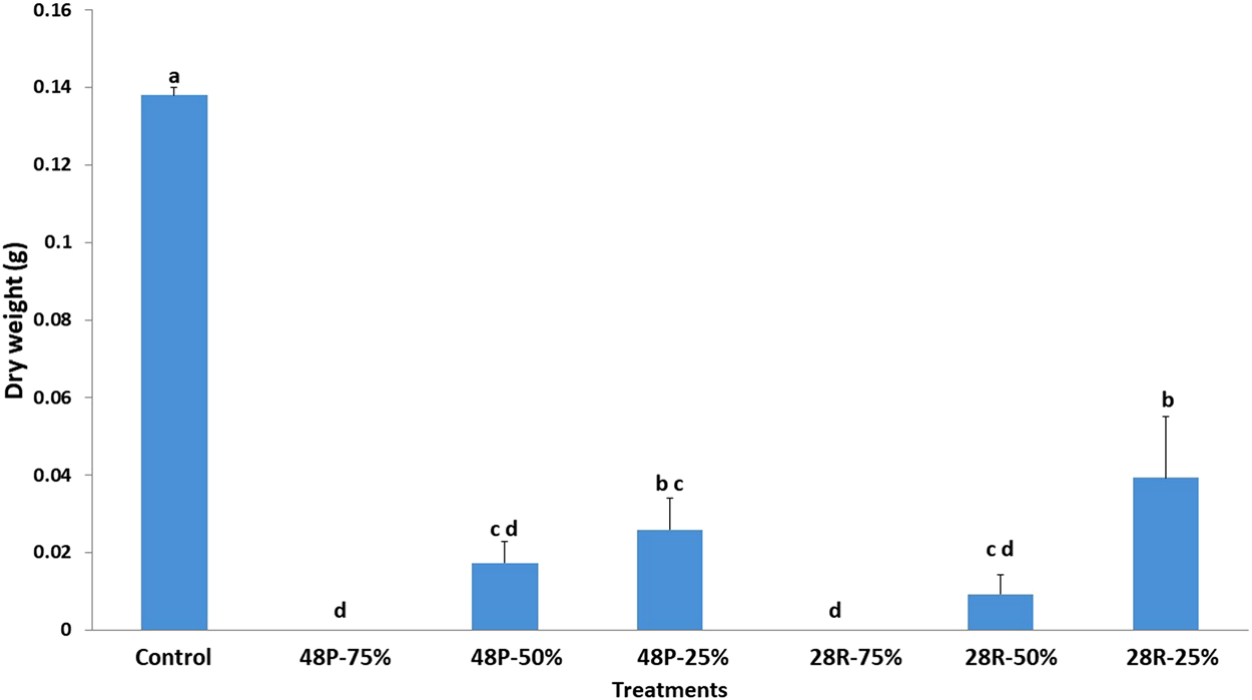
Influence of three different concentrations of *T*. *variabilis* culture filtrates (75%, 50% and 25%) on *P*. *aphanidermatum* growth. Columns and bars represent means ± SD.

Treating *P*. *aphanidermatum* with culture filtrates of *T*. *variabilis* isolates led to considerable inhibition in mycelial growth in all the tested concentrations (Fig. 2). *T*. *variabilis* isolates fully suppressed the growth of *P*. *aphanidermatum* at 75% concentration. However, the growth was reduced at 50% and 25% concentrations (Fig. 2). Furthermore, oospore production by *P*. *aphanidermatum* decreased significantly when it was exposed to 20% culture filtrate of *T*. *variabilis* (48 P: 8 oospores; 28 R: 9 oospores) compared to the control (56 oospores) (Table 1).

### Extracellular conductivity

Addition of 48 P and 28 R culture filtrates to *P*. *aphanidermatum* mycelium resulted in an increase in the extracellular conductivity values to 21.56 mV and 13.66 mV respectively, compared to PDB control (0.86 mV) (Table 1).

### Glucanase activity of *T*. *variabilis* culture filtrates

The concentration of glucanase enzyme produced by both *T*. *variabilis* isolates were similar: 8.09 for 48 P and 8.21 for 28 R (Table 1). The enzyme activity is expressed in nmoles substrate consumed h^−1^ ml of culture filtrate.

### Determination of cellulase activity using filter paper assay (FPA)

Both isolates of *T*. *variabilis* strains had cellulase enzyme in their culture filtrates. Isolate 48 P had significantly higher concentration of cellulase activity, 2.12 μmol/min/ml compared to 28 R isolate, 0.43 μmol/min/ml (Table 1).

### Siderophore production by *T*. *variabilis* isolates

**Figure 3:**
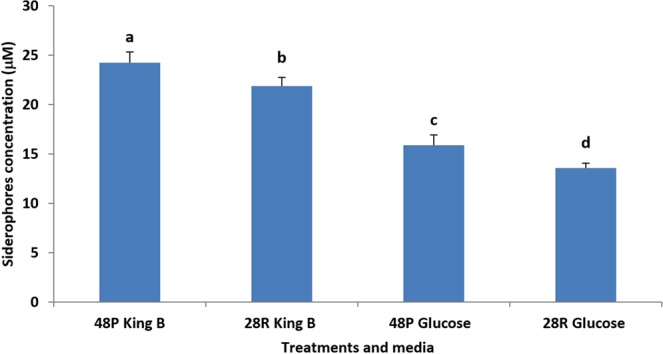
Siderophore production by *T*. *variabilis* isolates using King B and Glucose media. Columns and bars represent means ± SD.

Both isolates of *T*. *variabilis* produced siderophore in both media. However, King B medium contains the highest amount of siderophore, 24.25 μM for 48 P and 21.85 μM for 28 R compared to Glucose medium, 15.86 μM for 48 P and 13.56 μM for 28 R (Fig. 3).

### Effect of *T. variabilis* on *P. aphanidermatum* morphology

**Figure 4:**
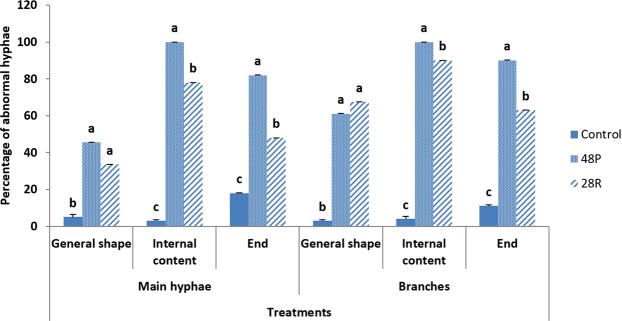
Effect of *T*. *variabilis* isolates 48 P and 28 R on *P*. *aphanidermatum* hyphal morphology compared to control. Columns and bars represent means ± CV of SD. Values with the same letters in the same category are not significantly different from each other (Pearson Chi-Square: asymptotic significance, 2-sided; >0.01).

Both isolates of *T*. *variabilis* induced significant abnormalities in general shape, internal content and the tips of main hyphae and hyphal branches of *P*. *aphanidermatum*. Isolate 48 P had a greater impact on hyphal morphology compared to isolate 28 R (Fig. 4).

**Figure 5:**
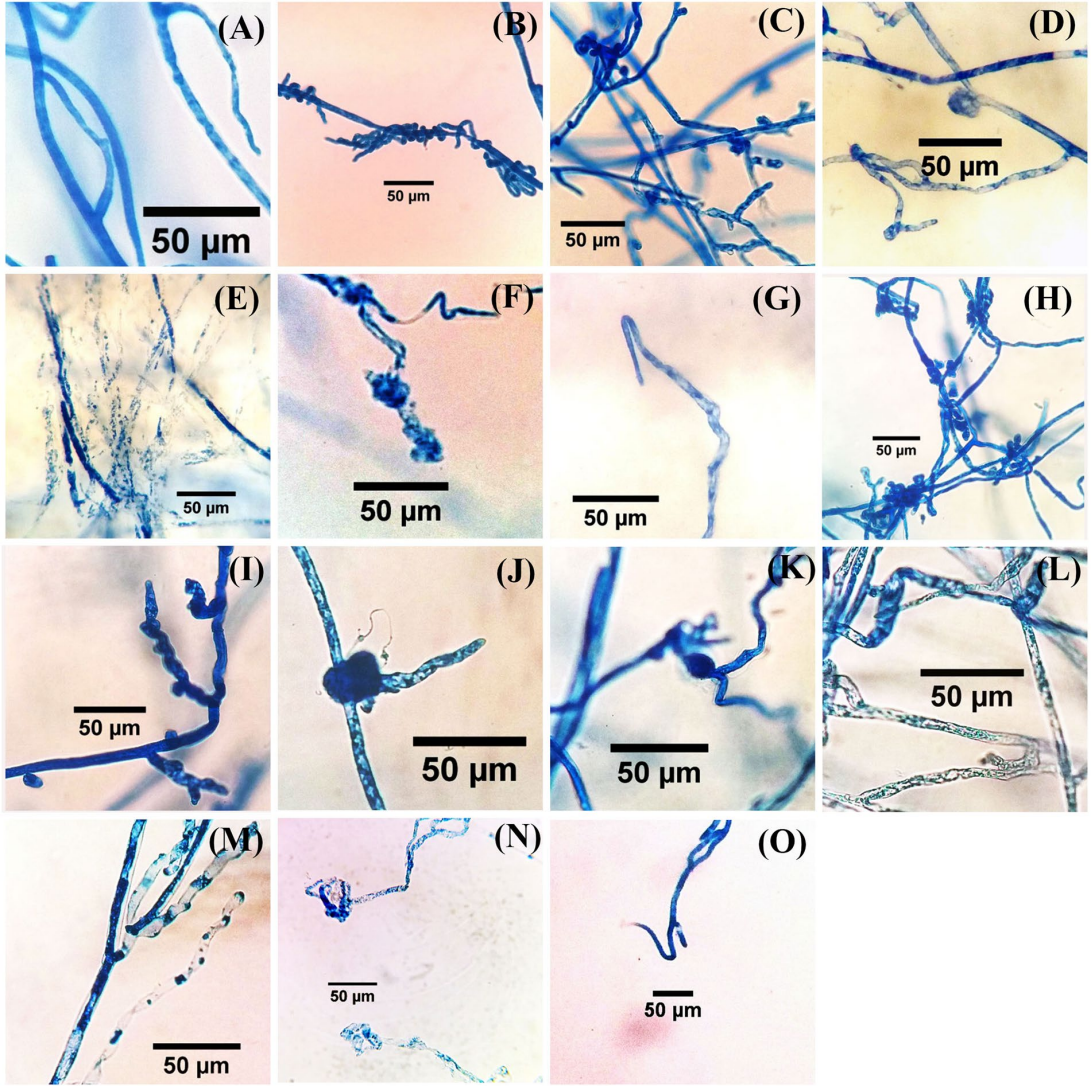
Abnormalities in hyphae of *P*. *aphanidermatum* under the effect of *T*. *variabilis* isolates using a light microscope. Normal hyphae of *P*. *aphanidermatum* (control; **A**); Effects of 48 P isolate on *P*. *aphanidermatum* hypha: twisted hyphae (**B**), hyphae with protrusions (**C**) loss of hyphal content (**D**), loss of internal content (**E**), wrapped up tip (**F**) and hook-like tip (**G**); Effects of 28 R isolate on *P*. *aphanidermatum* hypha: twisted (**H**), hyphae with protrusions and narrowings (**I**), hyphal content exit (**J**), bulbous hyphae (**K**), swollen hyphae (**L**), semi-empty internal content (**M**), wrapped-up tip (**N**) and hook-like tip (**O**). Scale bars = 50 μm.

Light microscope examination showed that the general shape of hypha became twisted, bulbous-like, swollen, loss of hyphal content and hyphae have protrusions and narrowings (Fig. 5). Also the internal content became empty or semi-empty, and the tips became wrapped-up, and wavy (Fig. 5).

**Figure 6:**
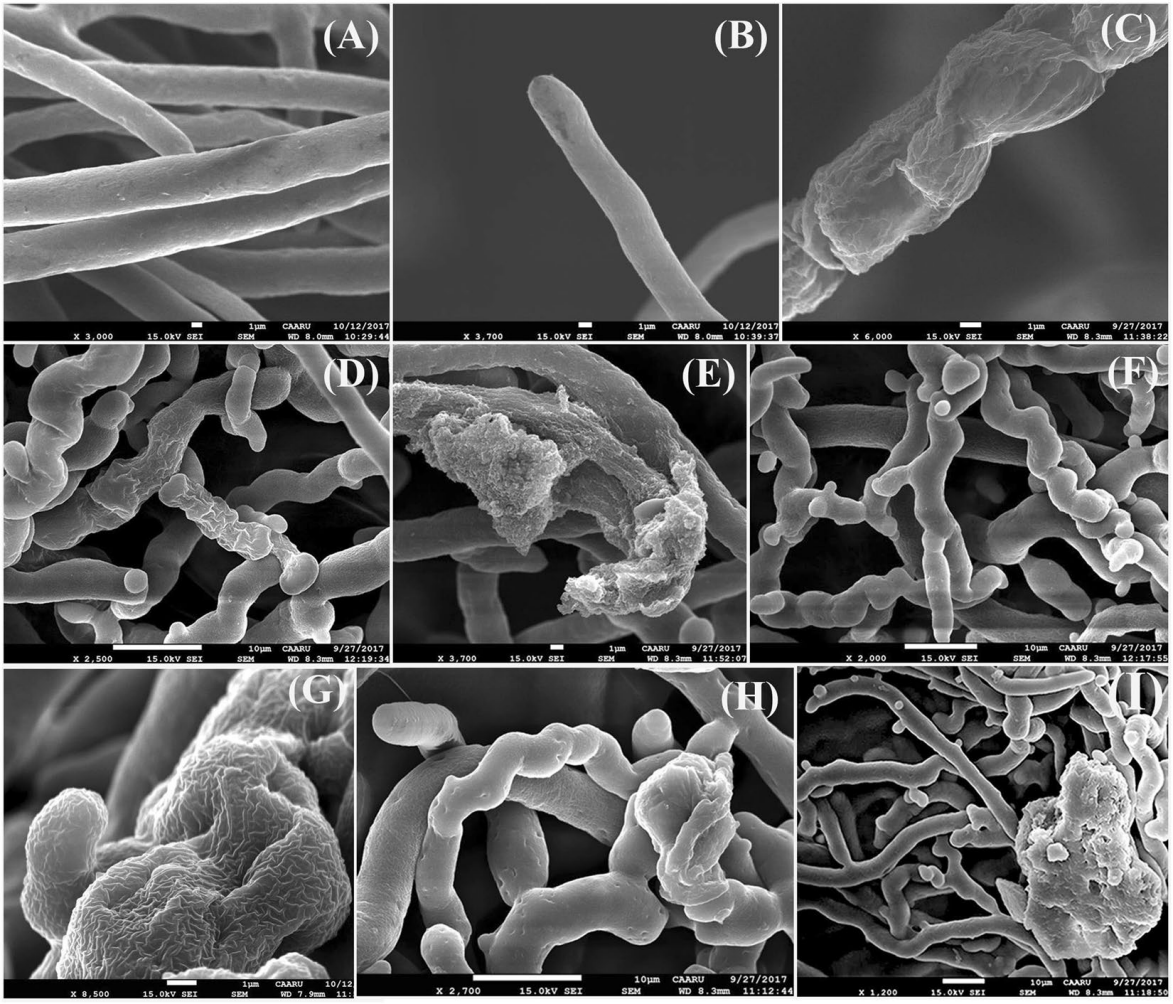
Abnormalities in hyphae of *P*. *aphanidermatum* under the effect of *T*. *variabilis* isolates using scanning electron microscope. Normal hyphae of *P*. *aphanidermatum* (control; **A**,**B**); Effects of 48 P isolate on *P*. *aphanidermatum* hypha: shrunken (**C**), wavy (**D**), loss of hyphal content (**E**) and hyphae with protrusions and narrowings (**F**); Effects of 28 R isolate on *P*. *aphanidermatum* hypha: shrunken (**G**), wavy (**H**) and loss of hyphal content and hyphae with protrusions (**I**).

Furthermore, scanning electron microscope showed similar observations such as shrunken and wavy hyphae, hyphal content exits and hyphae have protrusions and narrowings, as compared to the control which had straight, smooth surface and full hyphae (Fig. 6).

### Biocontrol potential of *T*. *variabilis* isolates against damping-off diseases of cucumber and tomato

*T*. *variabilis* isolates (48 P and 28 R) did not cause any significantly harmful effects on the length, fresh weight and dry weight of cucumber and tomato seedlings (Tables 2, 3). Both *T*. *variabilis* isolates 48 P and 28 R had considerable biocontrol efficacy against damping-off disease in cucumber and tomato (Table 3). Treating cucumber seeds with spores and mycelial suspension of *T*. *variabilis* isolate 48 P and *P*. *aphanidermatum* led to significant improvement in seedlings survival (51.78%) compared to the control (17.86%). Similar results were observed by *T*. *variabilis* isolate 28 R with significant improvement in cucumber survival (66.07%) compared to the control (30.36%) (Table 3).

**Table 2:** Effects of *T*. *variabilis* isolates on shoot length, shoot fresh weight and shoot dry weight of cucumbers and tomatoes.

	Isolate 48 P		Isolate 28 R	
	Control	Treatment	Control	Treatment
Cucumber shoot length (cm)	29.34 ± 1.77a	28.96 ± 2.05a	28.57 ± 2.6a	29.62 ± 2.74a
Tomato shoot length (cm)	19.33 ± 2.05a	21.86 ± 1.12a	19.33 ± 2.05a	20.71 ± 1.41a
Cucumber fresh shoot weight (g)	7.84 ± 0.87a	8.1 ± 0.94a	5.9 ± 0.85a	6.41 ± 1.01a
Tomato fresh shoot weight (g)	1.54 ± 0.43a	2.18 ± 0.38a	1.54 ± 0.43a	1.58 ± 0.31a
Cucumber dry shoot weight (mg)	0.572 ± 0.09a	0.573 ± 0.08a	0.358 ± 0.05a	0.362 ± 0.06a
Tomato dry shoot weight (mg)	0.114 ± 0.03a	0.193 ± 0.04a	0.11 ± 0.03a	0.13 ± 0.03a

Values with the same letters in the same row for each fungal treatment and its control are not significantly different from each other (ANOVA Test, P > 0.05). Values represent the mean ± SD.

**Table 3:** Biocontrol effect of *T*. *variabilis* isolates against damping-off disease caused by *P*. *aphanidermatum* in cucumbers and tomatoes.

Treatments	Cucumbers seedlings’ survival percentage		Tomatoes seedlings’ survival percentage	
	48 P	28 R	48 P	28 R
Control	94.64 ± 2.25a	83.93 ± 4.89a	83.93 ± 4.3a	83.92 ± 4.3a
Fungus	94.64 ± 2.25a	87.5 ± 2.78a	78.57 ± 4.64a	80.35 ± 3.23a
*P*. *aphanidermatum*	17.86 ± 5.06c	30.36 ± 8.51b	19.64 ± 4.6b	7.14 ± 3.28c
Fungus + *P*. *aphanidermatum*	51.78 ± 3.98b	66.07 ± 5.65a	64.28 ± 4.64a	35.71 ± 5.19b

Values with the same letters are not significantly different from each other (Duncan test, P > 0.05).Values represent the percentage ± 95% confidence limit.

Similarly, treating tomato seeds with spore and mycelial suspension of 48 P and *P*. *aphanidermatum* led to significant improvement in seedling survival (64.28%) compared to the control (19.64%). Also, 28 R significantly improved tomato survival (35.71%) compared to the control (7.14%).

## Discussion

Two strains of *T*. *variabilis* 48 P and 28 R were isolated from *Rhazya stricta* and *Zygophyllum coccineum*, respectively. The two plants are native to Oman. Species of *Talaromyces* are known endophytes and found on a wide range of plants such as *Potentilla fulgens*^24^, *Dactylis glomerata*^25^ and *Aloe vera*^26^. The current study proved that *T*. *variabilis* isolates were not pathogenic to cucumbers and tomatoes plants. This result was compatible with the definition of endophytes as fungi which colonize the stems and leaves of plants without causing any symptoms of disease^27^.

Our study clearly demonstrated that *T*. *variabilis* isolates produced inhibition zones against *P*. *aphanidermatum* on PDA media. Many other fungi such as *Trichoderma* spp.^28^ and bacteria such as *Pseudomonas fluorescens*^29^ are known to be antagosnistic against harmful pathogens such as *Aspergillus flavus*, *Fusarium moniliforme* and *Rhizoctonia solani*. The inhibition activity is mainly due to their ability to secrete bioactive compounds that inhibit plant pathogens^30^.

The culture filtrates of *T*. *variabilis* isolates were effective against *P*. *aphanidermatum* growth in liquid media. They significantly decreased *P*. *aphanidermatum* dry weight at 25% and 50% concentrations, and fully suppressed its growth at 75% concentration. Previous studies showed that *Streptomyces hydrogenans* culture filtrates inhibited the growth of *Alternaria Brassicicol*^31^. Oospore production was greatly decreased in the presence of culture filtrates of 48 P and 28 R. Similar observation was made by^16^, where *Aspergillus terreus* affected spore production by *P*. *aphanidermatum*.

Our data showed that both *Talaromyces* isolates produce cellulase enzyme. Cellulase enzyme can be produced by several fungal genera such as *Trichoderma*^32^, *Aspergillus*^33,34^ and *Talaromyces*^35^. Fungi and bacteria that secrete hydrolytic enzyme have biocontrol ability against plant pathogen. For example chitinase, glucanase and protease enzymes produced by *Trichoderma harzianum* are antagonistic against some fungi^36^. Chitinase and β-1,3-glucanase enzymes produced by *Clonostachys rosea* f. *catenulata* were responsible for efficient biocontrol against fungal plant pathogens^37^. About 18% of the *P*. *aphanidermatum* cell wall consists of cellulose^38^. The efficacy of *Talaromyces* isolates as biocontrol agents against damping-off disease may be in-part due to cellulose production by *Talaromyces* isolates.

Loss in integrity of *P*. *aphanidermatum* cells was observed due to *T*. *variabilis* isolates 48 P and 28 R culture filtrates. Consistently, the antifungal metabolites produced by *Sporothrix flocculosa* led to cellular leakage in several phytopathogens^39^. Another study by Zhao, *et al*.^40^ showed that *Streptomyces bikiniensis* causes cellular leakage in *Fusarium oxysporum*.

Our study showed the production of glucanase enzyme by 48 P and 28 R isolates. Several previous studies also documented a role of extracellular enzymes in biocontrol of pathogens. Examples include cellulases produced by *Lysinibacillus sphaericus*^19^ and chitinases and glucanases produced by *Trichoderma* species^41^. Our results showed that siderophores were produced by 48 P and 28 R isolates in King B and glucose media. Numerous fungi and bacteria could produce siderophores that are effective against pathogens. Siderophores produced by *Rhizobium meliloti* led to inhibition of *Macrophomina phaseolina*^42^. Moreover, cucumbers damping-off disease was controlled by *Aspergillus terreus* which was able to produce siderophores^16^. These siderophores may deprive the pathogen of iron, thus limiting essential nutrient^43^.

The morphology of *P*. *aphanidermatum* hyphae were significantly affected by 48 P and 28 R isolates, showing several abnormalities. Getha and Vikineswary^44^ showed the following abnormalities in *Fusarium oxysporum* hypha due to antagonistic influence of *Streptomyces violaceusniger*: swelling, distortion and excessive branching of hyphae, thickened with bulbous-like formation along the ends. Another study by Halo, *et al*.^16^ showed abnormalities in hyphae of *P*. *aphanidermatum* such as shrunken, semi empty and empty content and wrapped up ends under the effect of *Aspergillus terreus*.

Our study confirmed the efficient role of 48 P and 28 R isolates against cucumbers damping-off and tomatoes damping-off diseases. A previous study by Sivan, *et al*.^45^ showed the suppression *P*. *aphanidermatum* by *Trichoderma harzianum*. Similarly, *Gliocladium catenulatum* inhibited cucumbers damping-off and root rot diseases caused by *P*. *aphanidermatum*^46^. Also, damping-off of tomatoes disease caused by *P*. *aphanidermatum* was inhibited by *Trichoderma viride* and *Pseudomonas fluorescens* biocontrol agents^47^. Furthermore, endophytic actinomycetes were able to supress pathogenic activities of *P*. *aphanidermatum* because they produce glucanase enzyme^48^.

Our study is the first comprehensive report on the efficacy of *T*. *variabilis* isolates and their byproducts on *P*. *aphanidermatum* and Pythium damping-off of cucumbers and tomatoes.

The efficacy of these endophytes in suppressing *P*. *aphanidermatum* in the *in vitro* and *in vivo* tests through multiple mechanisms suggests that they may be effective against other harmful phytopathogens, including Pythium species that cause diseases in other plants. Also, using these endophytes provide an efficient alternative to the use of synthetic chemicals because P. aphanidermatum is less likely to develop resistance against these antagonistic fungi because they have multiple modes of action.

## Materials and Methods

### *Talaromyces* isolates

*Rhazya stricta* and *Zygophyllum coccineum* plants from desert sites in the Sultanate of Oman were selected for the isolation of endophytic fungi. The collections of samples was in May-August 2016 from Adam, 150 km from Muscat, the capital area of Oman. The method of Larran, *et al*.^49^ was followed for endophytic fungi isolation, as described by Halo *et al*.^16^.

Two fungal isolates (*Talaromyces*) were identified using sequences of three genes: the internal transcribed spacer region of the ribosomal RNA (ITS), beta-tubulin (TUB) and Calmodulin (CMD). The three genes were amplified using ITS1/ITS4, BT2A/BT2B and CMD5/CMD6 primers, respectively, using previously described reaction mixtures and conditions^50–52^. MEGA V.6 was used for sequence alignment^53^. Sequences were deposited in GenBank under accession numbers: ITS (48 P: MG957181, 28 R: MH006605), TUB (48 P: MH000341, 28 R: MH006606) and CMD: (48 P: MG979054, 28 R: MH006607).

### Antagosnistic effect of *T*. *variabilis* isolates against *P*. *aphanidermatum*

The antagosnistic acivity of *T*. *variabilis* isolates 48 P and 28 R against *P*. *aphanidermatum* was investigated using fresh cultures of *P*. *aphanidermatum* and *T*. variabilis as explained in our previous work^54^, using dual culture assay. There were four replicates in each experiment.

Isolates 48 P and 28 R were grown on PDB media in an incubator shaker at 28 °C for ten days to produce effective metabolites. The culture filtrates were obtained by centrifugation at 10,000 g, filtered through 0.2 µm Minisart filters, transferred to conical flasks and stored at 4 °C for further experiments.

Three concentrations of culture filtrates (75%, 50% and 25%) were used to study their effect on *P*. *aphanidermatum* growth in PDB media while the control included only PDB media. The concentration 75% consisted of 75% filtrate and 25% PDB, and so on for the other concentrations. A 3-mm diameter disk of *P*. *aphanidermatum* was added to each flask. Flasks were then kept in an incubator shaker at 28 °C and 120 rpm for 7 days. Finally, the liquid was disposed and the mycelium of *P*. *aphanidermatum* was dried in an oven at 65 °C for 24 h. The dry weights of the treatments and control were measured using three replicates for each isolate.

### Effect of culture filtrates of *T*. *variabilis* on extracellular conductivity and oospore production by *P*. *aphanidermatum*

The leakage of cellular components from mycelium of *P*. *aphanidermatum* was studied using 5 mg of dried mycelium obtained from the liquid culture. 10 ml of *T*. *variabilis* culture filtrates was added to dried mycelium and centrifuged. Extracellular conductivity of the culture was measured after 24 h^16,31^.

The effect of *T*. *variabilis* on oospore production of *P*. *aphanidermatum* was studied using V8 agar medium with 20% of *T*. *variabilis* culture filtrate. There were three replicates per treatment. The V8 agar medium without *T*. *variabilis* culture filtrate served as control^16^.

### Glucanase activity of *T*. *variabilis* culture filtrates

Glucanase production by *T*. *variabilis* isolates was detected using a protocol described by Jackson, *et al*.^55^. Samples were analyzed spectrophotometrically at 400 nm using an ELISA spectrophotometer. The final enzyme activity was calculated as per Jackson, *et al*.^55^.

### Cellulase activity

Two *T*. *variabilis* strains were cultivated in basal medium of Mandels and Weber (1969)^56^ supplemented with 1% cellulose. The flasks were incubated in a rotary shaker at 200 rpm at 28 °C for 10 days. Culture filtrates were obtained through centrifugation and used fresh. Total cellulase activities of fungal strains were determined as described by Mandel and Sternberg (1976)^57^.

### Siderophore production by *T*. *variabilis*

Based on our previous experiment^16^, the highest concentrations of siderophore were obtained using King B medium and glucose medium. The media were inoculated with disks from fresh PDA cultures of 48 P and 28 R and kept in an incubator shaker at 120 rpm at 28 °C for 7 days. The supernatants were obtained by centrifugation followed by filtration through 0.2 µM Minisart filters. ELISA spectrophotometer at 400 nm was used to detect siderophores concentrations using molar extinction coefficient *ε* = 20000^58,59^. The experiment had six replicates.

### Effect of *T. variabilis* on morphology of *P. aphanidermatum*

The antagonistic activity of *T*. *variabilis* isolates (48 P and 28 R) was checked against *P*. *aphanidermatum in vitro* as detailed in Halo, *et al*.^16^. The morphological study focused on main hyphae and their tips. 50 main and hypha tips of *P*. *aphanidermatum* near the inhibition zone were examined using a light microscope and scanning electron microscope (SEM, INSTUMENT JSM- 5600). These hyphae were compared with *P*. *aphanidermatum* hyphae in PDA control plate. All hyphae were stained with cotton blue.

### Biocontrol potential of *T*. *variabilis* isolates against damping-off diseases of cucumber and tomato

The effect of *T*. *variabilis* isolates (48 P and 28 R) on *Pythium* damping-off of cucumber and tomato was tested using three treatments and one control. 48 P and 28 R isolates, used in the experiment, were grown in PDB media in an incubator shaker at 28 °C and 120 rpm for 15 days to obtain fungal suspension (spore/mycelial). Also, this test used fresh cultures of *P*. *aphanidermatum* which were grown in PDA for 3 days at 28 °C. Seven sterilised seeds of cucumber or tomato were sown in each pot. Pots (12-cm in diameter) were autoclaved once. However, soil used in the pots was autoclaved twice.

Four replicate pots were kept for each treatment or control. The control pots were irrigated with 25 ml PDB media; the pots in first treatment were inoculated with 25 ml of fungal suspension containing spore and/or mycelia of 48 P or 28 R; the second treatment was treated with full plate of fresh PDA culture of *P*. *aphanidermatum*, 2 cm below soil surface; the pots in the third treatment was treated with full plate of fresh PDA culture of *P*. *aphanidermatum* and 25 ml of 48 P or 28 R fungal suspension^60^. The experiment was conducted at 28 °C for three weeks at 12–14 hr day length. After that, shoot length, fresh weight and dry weight were determined. Also seedlings survival rate was calculated by dividing the number of surviving seedlings by 7 (total seed sown) and then multiplying them by 100. This experiment was executed twice.

### Statistical analysis

Data were analysed by IBM SPSS Statistics 24.0 using Chi-Square test for morphological study to compare performance of the isolates to the control. The Poisson test was used to compare oospore production by *P*. *aphanidermatum* treated with each of the culture filtrates. Independent sample t-test, One-way ANOVA and Duncan’s Multiple Range Test were used to compare means of the different treatments. Each test is explained in the caption of each figure/table in the results section.

## Data Availability

All data underlying this publication are available in the manuscript.

## References

[1] Al-Sa’di AM (2008). First report of *Pythium splendens* associated with severe wilt of muskmelon (*Cucumis melo*) in Oman. Plant Disease.

[2] Stanghellini ME, Phillips JM (1975). *Pythium aphanidermatum*: its occurrence and control with pyroxychlor in the Arabian desert at Abu Dhabi. Plant Disease Reporter.

[3] Kipngeno P, Losenge T, Maina N, Kahangi E, Juma P (2015). Efficacy of Bacillus subtilis and Trichoderma asperellum against Pythium aphanidermatum in tomatoes. Biological Control.

[4] Lee S, Garzon CD, Moorman GW (2010). Genetic structure and distribution of *Pythium aphanidermatum* populations in Pennsylvania greenhouses basd on analysis of AFLP and SSR markers. Mycologia.

[5] Sharma P, Sain S (2005). Use of biotic agents and abiotic compounds against damping off of cauliflower caused by Pythium aphanidermatum. Indian Phytopathology.

[6] Weiland JE, Santamaria L, Grünwald NJ (2014). Sensitivity of Pythium irregulare, P. sylvaticum, and P. ultimum from forest nurseries to mefenoxam and fosetyl-Al, and control of Pythium damping-off. Plant Disease.

[7] Deadman, M., Al Maqbali, Y., Al Sa’di, A., Al Hasani, H. & Al Nabhani, M. In *Acta Horticulturae* Vol. 731, 367–370 (2007).

[8] Al-Sadi AM, Al-Masoodi RS, Al-Ismaili M, Al-Mahmooli IH (2015). Population structure and development of resistance to hymexazol among *Fusarium solani* populations from date palm, citrus and cucumber. Journal of Phytopathology.

[9] Xinxian L (2011). Isolation and characterization endophytic bacteria from hyperaccumulator Sedum alfredii Hance and their potential to promote phytoextraction of zinc polluted soil. World Journal of Microbiology and Biotechnology.

[10] Nath R, Sharma G, Barooah M (2015). Plant growth promoting endophytic fungi isolated from tea (Camellia sinensis) shrubs of Assam, India. Appl Ecol. Environ Res.

[11] Waqas M (2012). Endophytic fungi produce gibberellins and indoleacetic acid and promotes host-plant growth during stress. Molecules.

[12] Khan AL (2017). Bacillus amyloliquefaciens BSL16 improves phytoremediation potential of Solanum lycopersicum during copper stress. Journal of Plant Interactions.

[13] Li H-Y, Wei D-Q, Shen M, Zhou Z-P (2012). Endophytes and their role in phytoremediation. Fungal Diversity.

[14] Mejía LC (2008). Endophytic fungi as biocontrol agents of Theobroma cacao pathogens. Biological Control.

[15] Berg, G. & Hallmann, J. In *Microbial root endophytes* 53–69 (Springer, 2006).

[16] Halo BA, Al-Yahyai R, Al-Sadi AM (2018). Aspergillus terreus inhibits growth and induces morphological abnormalities in Pythium aphanidermatum and suppresses Pythium-induced damping-off of cucumber. Frontiers in Microbiology.

[17] Stosz SK, Fravel DR, Roberts DP (1996). *In vitro* analysis of the role of glucose oxidase from Talaromyces flavus in biocontrol of the plant pathogen Verticillium dahliae. Applied and environmental microbiology.

[18] Madi L, Katan T, Katan J, Henis Y (1997). Biological control of Sclerotium rolfsii and Verticillium dahliae by Talaromyces flavus is mediated by different mechanisms. Phytopathology.

[19] Naureen Z (2017). Exploring the potentials of Lysinibacillus sphaericus ZA9 for plant growth promotion and biocontrol activities against phytopathogenic fungi. Frontiers in microbiology.

[20] Buysens S, Heungens K, Poppe J, Hofte M (1996). Involvement of pyochelin and pyoverdin in suppression of Pythium-induced damping-off of tomato by Pseudomonas aeruginosa 7NSK2. Applied and Environmental Microbiology.

[21] Maurhofer M, Keel C, Haas D, Défago G (1994). Pyoluteorin production by Pseudomonas fluorescens strain CHA0 is involved in the suppression of Pythium damping-off of cress but not of cucumber. European journal of plant pathology.

[22] Schulz B, Boyle C, Draeger S, Römmert A-K, Krohn K (2002). Endophytic fungi: a source of novel biologically active secondary metabolites. Mycological Research.

[23] Singh LP, Gill SS, Tuteja N (2011). Unraveling the role of fungal symbionts in plant abiotic stress tolerance. Plant signaling & behavior.

[24] Bhagobaty R, Joshi S (2011). Fungal endophytes of five medicinal plants prevalent in the traditionally preserved ‘Sacred forests’ of Meghalaya, India. Forest Science and Technology.

[25] Márquez SS, Bills GF, Zabalgogeazcoa I (2007). The endophytic mycobiota of the grass Dactylis glomerata. Fungal Divers.

[26] Bara R (2013). Talaromins A and B, new cyclic peptides from the endophytic fungus Talaromyces wortmannii. Tetrahedron Letters.

[27] Carroll G (1988). Fungal endophytes in stems and leaves: from latent pathogen to mutualistic symbiont. Ecology.

[28] Calistru C, McLean M, Berjak P (1997). *In vitro* studies on the potential for biological control of Aspergillus flavus and Fusarium moniliforme by Trichoderma species. Mycopathologia.

[29] Nagarajkumar M, Bhaskaran R, Velazhahan R (2004). Involvement of secondary metabolites and extracellular lytic enzymes produced by Pseudomonas fluorescens in inhibition of Rhizoctonia solani, the rice sheath blight pathogen. Microbiological Research.

[30] Bernal G, Illanes A, Ciampi L (2002). Isolation and partial purification of a metabolite from a mutant strain of Bacillus sp. with antibiotic activity against plant pathogenic agents. Electronic Journal of Biotechnology.

[31] Manhas RK, Kaur T (2016). Biocontrol potential of Streptomyces hydrogenans strain DH16 toward Alternaria brassicicola to control damping Off and black leaf spot of Raphanus sativus. Frontiers in plant science.

[32] de Souza MF, da Silva ASA, Bon EP (2018). A novel Trichoderma harzianum strain from the Amazon Forest with high cellulolytic capacity. Biocatalysis and Agricultural. Biotechnology.

[33] Mohapatra, S., Padhy, S., Mohapatra, P. K. D. & Thatoi, H. Enhanced reducing sugar production by saccharification of lignocellulosic biomass, Pennisetum species through cellulase from a newly isolated Aspergillus fumigatus. *Bioresource Technology* (2018). 10.1016/j.biortech.2018.01.023 29353755

[34] Zhao C, Deng L, Fang H (2018). Mixed culture of recombinant Trichoderma reesei and Aspergillus niger for cellulase production to increase the cellulose degrading capability. Biomass and Bioenergy.

[35] Jain, L., Kurmi, A. K. & Agrawal, D. Conclusive selection of optimal parameters for cellulase production by Talaromyces verruculosus IIPC 324 under SSF via saccharification of acid-pretreated sugarcane bagasse. *Biofuels*, 1–9 (2018).

[36] Schirmböck M (1994). Parallel formation and synergism of hydrolytic enzymes and peptaibol antibiotics, molecular mechanisms involved in the antagonistic action of Trichoderma harzianum against phytopathogenic fungi. Applied and Environmental Microbiology.

[37] Chatterton S, Punja ZK (2009). Chitinase and β-1, 3-glucanase enzyme production by the mycoparasite Clonostachys rosea f. catenulata against fungal plant pathogens. Canadian journal of microbiology.

[38] Blaschek W, Käsbauer J, Kraus J, Franz G (1992). Pythium aphanidermatum: culture, cell-wall composition, and isolation and structure of antitumour storage and solubilised cell-wall (1→ 3),(1→ 6)-β-d-glucans. Carbohydrate research.

[39] Hajlaou M, Traquair J, Jarvis W, Bélanger R (1994). Antifungal activity of extracellular metabolites produced by Sporothrix flocculosa. Biocontrol Science and Technology.

[40] Zhao S, Du C-M, Tian C-Y (2012). Suppression of Fusarium oxysporum and induced resistance of plants involved in the biocontrol of Cucumber Fusarium Wilt by Streptomyces bikiniensis HD-087. World Journal of Microbiology and Biotechnology.

[41] Howell C (2003). Mechanisms employed by Trichoderma species in the biological control of plant diseases: the history and evolution of current concepts. Plant disease.

[42] Arora, N., Kang, S. & Maheshwari, D. Isolation of siderophore-producing strains of Rhizobium meliloti and their biocontrol potential against Macrophomina phaseolina that causes charcoal rot of groundnut. *Current Science*, 673–677 (2001).

[43] Dowling DN, O’Gara F (1994). Metabolites of Pseudomonas involved in the biocontrol of plant disease. Trends in Biotechnology.

[44] Getha K, Vikineswary S (2002). Antagonistic effects of Streptomyces violaceusniger strain G10 on Fusarium oxysporum f. sp. cubense race 4: indirect evidence for the role of antibiosis in the antagonistic process. Journal of Industrial Microbiology and Biotechnology.

[45] Sivan A, Elad Y, Chet I (1984). Biological control effects of a new isolate of Trichoderma harzianum on Pythium aphanidermatum. Phytopathology.

[46] Punja ZK, Yip R (2003). Biological control of damping-off and root rot caused by *Pythium aphanidermatum* on greenhouse cucumbers. Canadian. Journal of Plant Pathology.

[47] Manorantitham S, Prakasam V, Rajappan K (2001). Biocontrol of damping off of tomato caused by Pythium aphanidermatum. Indian Phytopathology.

[48] El‐Tarabily K, Nassar A, Hardy GSJ, Sivasithamparam K (2009). Plant growth promotion and biological control of Pythium aphanidermatum, a pathogen of cucumber, by endophytic actinomycetes. Journal of Applied Microbiology.

[49] Larran S, Perello A, Simon M, Moreno V (2002). Isolation and analysis of endophytic microorganisms in wheat (Triticum aestivum L.) leaves. World Journal of Microbiology and Biotechnology.

[50] White, T. J., Bruns, T., Lee, S. & Taylor, J. In *PCR protocols: A Guide to Methods and Applications* (eds Innis, M. A., Gelfand, D. H., Sninsky, J. J. & White, T. J.) 315–322 (Academic Press, 1990).

[51] Koenraadt H, Somerville SC, Jones A (1992). Characterization of mutations in the beta-tubulin gene of benomyl-resistant field strains of Venturia inaequalis and other plant pathogenic fungi. Phytopathology.

[52] Hong S-B, Go S-J, Shin H-D, Frisvad JC, Samson RA (2005). Polyphasic taxonomy of Aspergillus fumigatus and related species. Mycologia.

[53] Tamura K, Stecher G, Peterson D, Filipski A, Kumar S (2013). MEGA6: Molecular Evolutionary Genetics Analysis version 6.0. Molecular Biology and Evolution.

[54] Halo B, Al-Yahyai R, Al-Sadi AM (2018). Aspergillus terreus inhibits growth and induces morphological abnormalities in Pythium aphanidermatum and suppresses Pythium-induced damping-off of cucumber. Frontiers in Microbiology.

[55] Jackson CR, Tyler HL, Millar JJ (2013). Determination of microbial extracellular enzyme activity in waters, soils, and sediments using high throughput microplate assays. Journal of visualized experiments: JoVE.

[56] Mandel M, Weber J (1969). Exoglucanase activity by microorganisms. Advance Chemistry.

[57] Mandel M, Sternberg D (1976). Recent advances in cellulose technology. J. Ferment. Technol.

[58] Meyer J (1978). a. & Abdallah, M. The fluorescent pigment of Pseudomonas fluorescens: biosynthesis, purification and physicochemical properties. Microbiology.

[59] Rachid D, Ahmed B (2005). Effect of iron and growth inhibitors on siderophores production by Pseudomonas fluorescens. African Journal of Biotechnology.

[60] Al-Hinai AH (2010). Isolation and characterization of *Pseudomonas aeruginosa* with antagonistic activity against *Pythium aphanidermatum*. Journal of Plant Pathology.

